# *RHOBTB2*-Associated Neurological Phenotypes and Underlying Mechanisms: Alternating Hemiplegia of Childhood Beyond *ATP1A3*

**DOI:** 10.3390/diseases14050166

**Published:** 2026-05-09

**Authors:** Ruzica Kravljanac, Kristel Klaassen, Vladimir Oparnica, Biljana Vucetic Tadic, Marina Andjelkovic, Anita Skakic, Sara Stankovic, Maja Stojiljkovic

**Affiliations:** 1 Institute for Mother and Child Healthcare of Serbia “Dr Vukan Cupic”, 11070 Belgrade, Serbia; 2 Faculty of Medicine, University of Belgrade, 11000 Belgrade, Serbia; 3 Institute of Molecular Genetics and Genetic Engineering, University of Belgrade, 11042 Belgrade, Serbia

**Keywords:** alternating hemiplegia of childhood (AHC), *RHOBTB2*, neurodevelopmental, phenotype, variant

## Abstract

Alternating hemiplegia of childhood (AHC) represents a severe and complex pediatric neurodevelopmental disorder, predominantly characterized by the occurrence of paroxysmal episodes of transient unilateral or bilateral paresis prior to 18 months of age. It belongs to a group of ultra-rare neurological disorders with a prevalence from 1:100,000 to 1:1,000,000. Even though the majority of AHC patients harbor pathogenic variants in the *ATP1A3* gene, recent studies have pinpointed to other causative genes in *ATP1A3*-negative patients, including *RHOBTB2*. In this review, we report the case of a patient with a severe phenotype of AHC associated with developmental delay, aphasia and epilepsy, caused by a pathogenic de novo variant in the *RHOBTB2* gene. Furthermore, we contribute a literature review on *ATP1A3*-negative AHC with a special focus on *RHOBTB2*-related AHC phenotypes, along with an overview of the pathophysiological mechanism of variants affecting residues in the BTB domain of the RHOBTB2 protein. The results of our study indicate that *RHOBTB2*-related AHC might have a more severe clinical presentation compared to *ATP1A3*-related AHC. Variants in the *RHOBTB2* gene should be considered as disease-causing in patients with early-onset seizures, delayed psychomotor development and alternating hemiplegia of childhood.

## 1. Introduction

Alternating hemiplegia of childhood (AHC) represents a complex neurodevelopmental disorder predominantly characterized by paroxysmal transient unilateral or bilateral paresis, with onset prior to 18 months of age [1]. It belongs to a group of rare, even ultra-rare neurological disorders with a prevalence from 1:100,000 to 1:1,000,000 [2,3]. The main criteria for AHC diagnosis were established by Aicardi et al. [4], defining a set of clinical features required for the diagnosis and aiming to distinguish it from other causes of episodic hemiplegia in childhood. According to them, paroxysmal hemiplegia was present in all cases, with episodes of double hemiplegia or quadriplegia in 93% of them, whereas other reported clinical manifestations included tonic and dystonic attacks present in 96% patients, neurological deficits, choreoathetosis or dystonia in 90% patients, and oculomotor abnormalities, such as nystagmus, gaze deviation or strabismus, in 97% cases. Developmental delay or intellectual disability was found in nearly all cases (98%). The remission of symptoms with sleep was observed in 94% cases [4].

Recently, revised diagnostic criteria for AHC were published, as previous guidelines have evolved to incorporate advances in clinical understanding and novel discoveries in the genetics of this disorder [5]. Namely, the *ATP1A3* gene, encoding the catalytic α3 subunit of neuronal Na^+^/K^+^ ATPases, was revealed as the cause of AHC, where pathogenic variants in this gene were found in 78–92% patients [6,7]. The identification of a predominant genetic cause has not only provided vital insight into the underlying pathophysiology of this disorder, but it has also completely transformed diagnostic approaches, emphasizing the impact of genetics and setting the stage for novel targeted therapeutic strategies.

Nevertheless, given that not all patients with AHC harbor variants in the *ATP1A3* gene, further studies were pursued to shed more light on the molecular basis of this disease. Panagiotakaki E et al. [8] evaluated a cohort of 26 ATP1A3-negative patients with AHC and discovered pathogenic and likely pathogenic variants in various neurodevelopmental genes, including *ATP1A2*, *RHOBTB2*, *ANK3*, *SCN2A*, and *CHD2*. One of the novel candidate genes for AHC proposed by Panagiotakaki et al. [8] was *RHOBTB2*, with three patients harboring de novo variants, of which two presented with typical AHC and one with atypical presentation, lacking obvious bouts of hemiplegia or quadriplegia. This gene was previously linked to AHC in a study by Zagaglia et al. [9], where they examined a cohort of patients with *RHOBTB2*-related disorders presenting with the phenotype of a complex movement disorder that resembled AHC.

The *RHOBTB2* gene, located on chromosome 8p21.3 and comprising nine exons, codes for Rho-related BTB domain-containing protein 2 (RhoBTB2), a small atypical Rho GTPase from the BTB domain-containing protein family. This protein plays an important role in numerous cellular processes, including cell polarity and migration, cell cycle regulation and regulation of cytoskeleton dynamics [10]. Also known as DBC2 (deleted in breast cancer 2), it was initially described and studied as a tumor suppressor [11], while the first association with neurodevelopmental disorders was not until 2018, when Straub et al. [12] discovered that pathogenic variants in the *RHOBTB2* gene cause a novel type of developmental and epileptic encephalopathy (DEE) (DEE64 (MIM: 618,004)).

Since the initial discovery of the link between *RHOBTB2* and the neurodevelopmental disorder, further reports emerged about patients with pathogenic variants in this gene. Individual case reports, as well as small groups of patients all around the world, added more information about the nature of the variants [13,14]. The phenotypic spectrum has also been widening, adding acute encephalopathy after head trauma [15] and excessive laughter [16], along with the first reported symptoms comprising early-onset seizures, encephalopathy, severe developmental delay, postnatal microcephaly and different movement disorders [12,17]. According to a recent comprehensive report by Langhammer et al. [18], only around 70 patients have been reported worldwide. Interestingly, this study did not report cases with AHC as the primary clinical feature (even though two patients presented with hemiplegia after seizures), so *RHOBTB2* phenotypes encompassing movement disorders, which include AHC, proved to be very rare. In this review, we report the case of a severe phenotype of AHC caused by a pathogenic de novo variant in the *RHOBTB2* gene. Furthermore, we contribute a literature review on *ATP1A3*-negative AHC with a special focus on *RHOBTB2*-related AHC phenotypes, along with an elaborated pathophysiological mechanism of disease-causing variants residing in the RHOBTB2 BTB domain.

## 2. Case Description

We present the clinical report of a female patient, born as the second child of healthy, nonconsanguineous parents, delivered vaginally at full term following an unremarkable pregnancy, with a birth weight of 3350 g and an Apgar score of 10. She had a developmental delay, presenting with hypotonia during infancy and a late onset of walking without support at 26 months. She has never developed speech, but she has nonverbal communication and good emotional engagement with other people.

The patient had her first attack at the age of 17 months. She was unresponsive and hypotonic, with leftward gaze deviation, horizontal nystagmus and left-sided hemiplegia. The duration of unresponsiveness was nearly three hours. Upon admission to the hospital, the patient experienced a generalized tonic-clonic seizure refractory to benzodiazepine therapy (intravenous diazepam and midazolam), which resolved following administration of intravenous phenobarbital at a dose of 20 mg/kg. During the second day of hospitalization, she had an attack with leftward gaze deviation and jerking of the limbs, so midazolam and phenobarbital were administered as rescue medications. Computerized tomography (CT) and brain magnetic resonance imaging (MRI) demonstrated bilateral myelination delay. Ictal electroencephalographic (EEG) recordings during two similar episodes described as jerking of the limbs lasting 110 and 128 s showed focal spike-waves and sharp-waves starting above the frontotemporal right region with spreading to the whole right hemisphere. Serial interictal EEG showed slow, high-amplitude activity of 2–3 Hz without epileptic discharges. Carbamazepine was introduced. She was febrile, with a temperature higher than 38 degrees Celsius, but without increased parameters for infection, and no signs of infection in her physical status. Slow recovery of left-sided hemiplegia was observed, and on the ninth day of hospitalization, the patient was discharged. At discharge, she was hypotonic with positive bilateral clonus and Babinski sign, and she was able to sit and walk with support. In the outpatient clinic during the following two months, clonus and Babinski were still present, but she had complete gross motor recovery. At an examination six months after discharge, no clonus and Babinski were noted, and a low dose of carbamazepine was maintained in the therapeutic regimen.

The second hospitalization was at the age of three years, due to right-sided hemiplegia provoked by a respiratory infection. During hospitalization, repeated deviations of the eyes toward one side and right-sided hemiparesis were noticed. Intravenous midazolam was administered, and the dosage of carbamazepine was increased up to 21 mg/kg. Since a clinical suspicion of ACH or other developmental and epileptic encephalopathies was raised, targeted genetic analysis was performed, but it came back negative, confirming the absence of pathogenic variants in the *ATP1A3* gene.

Over a two-year follow-up period, the patient exhibited recurrent episodes of hemiparesis, hemiplegia or quadriplegia, occurring at approximately six-month intervals, with variable duration (from hours to days) and alternating laterality. Introduction of flunarizine was considered and discussed with the parents, but the treatment was not started. The third hospitalization was at the age of four years due to sudden somnolence and quadriplegia. Interictal EEG showed slowing of background activity with no epileptic discharges. The dose of carbamazepine was corrected according to body weight (23 mg/kg), and the patient was discharged with significant motor improvement. The fourth hospitalization was at the age of 4.5 years due to right-sided hemiparesis lasting for hours. Interictal EEG showed slow delta and theta high amplitude activity above the left fronto-centrotemporal region. The fifth hospitalization was at the age of five years due to unresponsiveness, general hypotonia and motor weakness associated with a febrile episode. During hospitalization, the patient had repeated attacks with eye deviation upward and toward the right side, associated with jerking of the arms and left leg, which were terminated by diazepam and midazolam. Serial interictal EEG showed slow background activity (1–4 Hz), with spike-and-wave (SW) above the left centroparietal region. The sixth hospitalization was at the age of 5.5 years due to a short episode of unresponsiveness and deviation of the eyes during an acute febrile infection, with Streptococcus viridans detected in hemoculture. Neurological examination demonstrated right-sided hemiparesis, which progressively improved over the course of hospitalization. Over the following 2.5 years, the patient remained free of attacks.

**Figure 1:**
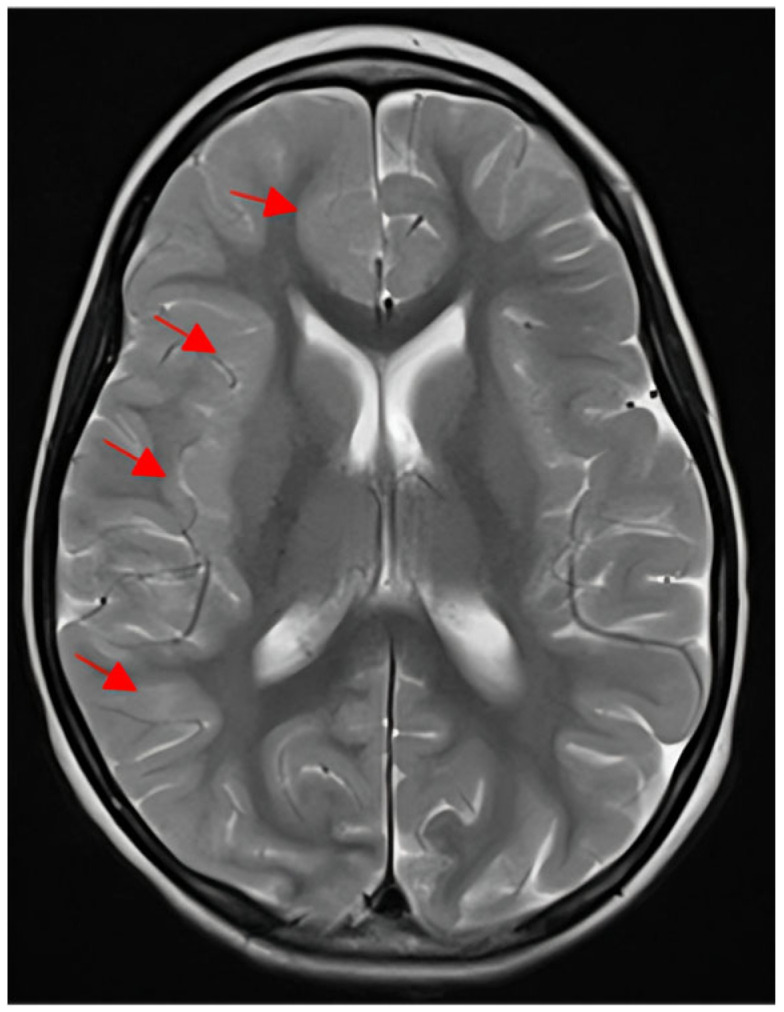
Brain MRI demonstrating bilateral myelination delay and right hemispheric cortical abnormalities (indicated with red arrows). MRI—magnetic resonance imaging.

The last hospitalization was at the age of seven years, due to sudden unresponsiveness, deviation of the eyes to the right side and left-side hemiplegia during a prolonged viral infection with a high fever. Frequent episodes of ocular deviation were observed throughout the day, persisting until the administration of diazepam. Although diazepam induced sleep, ocular deviation recurred upon the patient’s awakening. Diazepam was administered three to four times per day and was substituted with clobazam from the second day of hospitalization. Even though the frequency of ocular deviation episodes decreased with clobazam, the emergence of somnolence necessitated its discontinuation and the reintroduction of diazepam. Brain MRI conducted six days after the onset of left-sided hemiplegia demonstrated bilateral myelination delay, most prominent in the temporal lobes. Furthermore, right frontal and right parietal cortical thickening, blurring of the grey–white matter interface, and juxtacortical FLAIR hyperintensity were also observed (Figure 1).

**Figure 2:**
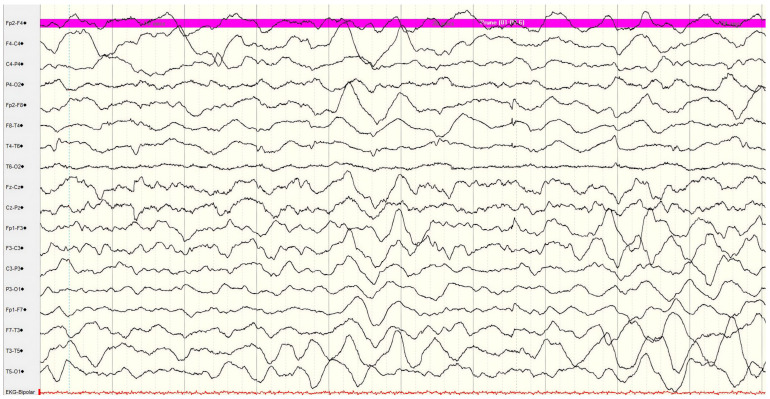
Interictal EEG during left-sided hemiplegia showing asymmetric background activity with right-side depression in amplitude and diffuse slow delta and theta activity. EEG—electroencephalography.

Interictal EEG showed asymmetric background activity with right-side depression in amplitude and diffuse slow delta and theta activity (Figure 2).

**Figure 3:**
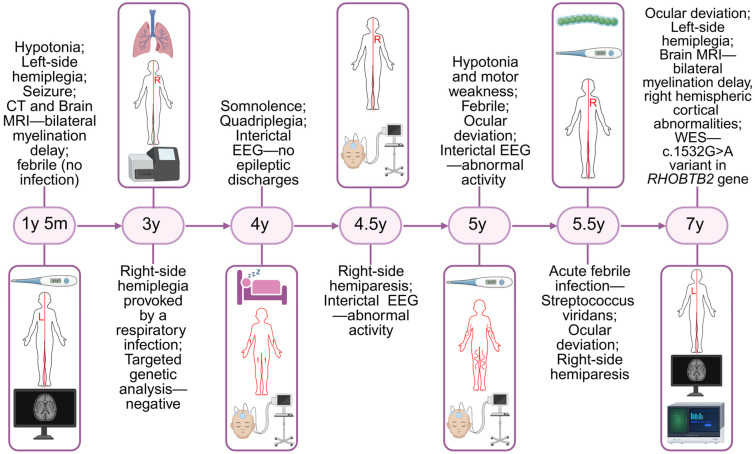
Timeline of major clinical events of the patient. CT—computerized tomography; WES—whole exome sequencing. Created in BioRender. Stankovic, S. (2026) https://BioRender.com/mmlm70h (accessed on 30 April 2026).

The clinical course was complicated by focal status epilepticus, manifesting as loss of consciousness, with ipsilateral hemifacial jerking propagating to the left upper and lower extremities, and a total seizure duration of two hours. Status epilepticus was treated successively by rectal diazepam and intravenous midazolam, phenobarbital (20 mg/kg) and levetiracetam (40 mg/kg), while in further course, corticosteroid therapy was introduced with dexamethasone. Following recovery from status epilepticus, the patient developed severe tonic crises. The episodes were characterized by progressive, generalized tonic posturing with associated upward gaze deviation, severe laryngeal spasm and inspiratory stridor. The crisis resulted in significant respiratory impairment, requiring endotracheal intubation due to life-threatening respiratory failure. The patient remained on artificial ventilation for three days, during which continuous midazolam infusion was maintained. In further course, the patient had slow improvement and became more active, although left-side hemiplegia persisted for more than two months. Neurological examination at the time of discharge revealed a nonverbal patient who demonstrated visual tracking, preserved responsiveness, left-sided central facial palsy, left-side hemiplegia with left leg clonus and absent Babinski sign. The patient was able to maintain a seated position with support but was unable to walk, even with assistance. During the subsequent follow-up period, intensive physical therapy resulted in significant recovery. After six months, no sign of hemiparesis was noted, and the patient has regained independent ambulation and returned to her pre-hospitalization neurological status. Adequate seizure control was achieved with levetiracetam at 50 mg/kg and carbamazepine at 23 mg/kg. Flunarizine was added upon discharge from the hospital and probably contributed to preventing the episodes of alternating hemiplegia since no episode was experienced during the following three years. Timeline of major clinical events of the patient is shown in Figure 3.

## 3. Genetic Analysis

Given that the targeted genetic analysis did not reveal disease-causing variants in the patient, we performed whole exome sequencing (WES) analysis at the Institute of Molecular Genetics and Genetic Engineering, Belgrade, as previously described [19]. Genomic DNA was isolated from whole blood using a QIAamp DNA Blood Mini Kit (Qiagen, Hilden, Germany) and further analyzed by WES. The library preparation was performed according to the BGI WES Library construction kit protocol, and sequencing was performed with a DNBSEQ-G400 sequencer (MGI Tech, Shenzhen, China), with alignment and analysis performed against the GRCh38/hg38 reference genome assembly. Systematic interpretation of variants was performed as follows: All variants were prioritized using VarSome Clinical (Saphetor, Lausanne, Switzerland) and classified according to the recommendations of the American College of Medical Genetics and Genomics [20]. In accordance with the clinical phenotype of our patient, genes associated with the following HPO terms were prioritized: neurodevelopmental delay HP:0012758, hypotonia HP:0001252, seizure HP:0001250, nystagmus HP:0000639, abnormality of eye movement HP:0000496, hemiplegia HP:0002301, hemiplegia/hemiparesis HP:0004374, and episodic hemiplegia HP:0012194. Segregation analysis and confirmation of the variant were performed using conventional Sanger sequencing, using the BigDye™ Terminator v3.1 Cycle Sequencing Kit on an Applied Biosystems SeqStudio™ Genetic Analyzer (Thermo Fisher Scientific, Waltham, MA, USA), with primers being available upon request. Whole exome sequencing revealed the presence of a missense heterozygous variant c.1532G>A (p.Arg511Gln) in the *RHOBTB2* gene. This genetic variant was classified as pathogenic (PS3 Very Strong, PM1 Moderate, PM5 Moderate, PM6 Moderate, PM2 Supporting, PP3 Supporting) according to the American College of Medical Genetics and Genomics (ACMG) classification, and it has been described in ClinVar (Variation ID: 545418) [20]. No other rare candidate variants that correlated highly with the clinical phenotype of the patient and met the inheritance pattern were identified in the exome sequencing data. Segregation analysis confirmed that the *RHOBTB2* variant c.1532G>A (p.Arg511Gln) was absent in both parental samples (Figure S1), consistent with a de novo occurrence, although formal verification of biological parentage was not performed.

## 4. Comprehensive Literature Search

A systematic literature search was conducted across three databases: PubMed, Google Scholar, and Europe PMC. The following search terms were applied: “*RHOBTB2* neurodevelopmental,” “*RHOBTB2* paroxysmal,” “*RHOBTB2* dyskinesia,” “*RHOBTB2* hemiplegia,” “*RHOBTB2* hemiplegic episode,” “*RHOBTB2* DEE,” “*RHOBTB2* alternating hemiplegia of childhood,” “*RHOBTB2* AHC,” and “*RHOBTB2* AHC-like.” No restrictions were applied to the publication date. The total number of articles retrieved per search term and per database is summarized in Table 1.

Only English-language publications were included. PhD theses, preprints, conference abstracts, and review articles without original case presentations were excluded. The remaining *RHOBTB2*-related publications were categorized into the following groups: DEE case presentations, AHC case presentations, Rett syndrome-like case presentations, case presentations of *RHOBTB2*-related disorders without epilepsy, and research articles without case presentations (Figure S2). All publications containing case presentations were subsequently screened for documented AHC cases associated with an underlying *RHOBTB2* variant. A total of 13 previously published cases met these criteria and were included in this review, together with one previously unpublished case described here in detail. In addition, research articles without individual case descriptions were also reviewed to provide a broader context for the functional impact of *RHOBTB2* variants, explore their potential association with the AHC phenotype, and support the discussion of possible underlying disease mechanisms. The literature search scheme is presented in the form of a flow diagram (Figure S3).

## 5. Discussion

### 5.1. Genetics of ATP1A3-Negative AHC

Alternating hemiplegia of childhood constitutes a severe and complex pediatric neurological disorder, the hallmark of which is the occurrence of paroxysmal episodes of transient unilateral or bilateral paresis in infancy. Ever since the initial research, which set the foundation for insights into the genetic basis of this disorder, AHC has been typically associated with pathogenic variants in the *ATP1A3* gene, which account for the majority of clinically well-defined cases. Nevertheless, a subset of patients fulfilling diagnostic criteria for AHC remains negative for *ATP1A3* variants, highlighting the genetic heterogeneity of the disorder. Recent sequencing studies have shown that these cases may be explained by variants in additional genes involved in neuronal excitability, ion transport, synaptic transmission, and brain energy metabolism. This supports the view of AHC as a clinically defined syndrome with partially overlapping molecular mechanisms, rather than a strictly single-gene disorder [5,8,21]. Table 2 presents the genetic spectrum of AHC led by the *ATP1A3* and *RHOBTB2* genes, as well as several less frequent or emerging genes associated with AHC-like phenotypes and relevant differential diagnoses.

Among the non-*ATP1A3* genes, the *SCN2A* gene currently has the strongest evidence as an alternative cause of AHC in *ATP1A3*-negative patients. In an exome sequencing study of *ATP1A3*-negative AHC cases, Panagiotakaki et al. [8] identified de novo *SCN2A* variants and concluded that variants in neurodevelopmental genes, particularly *SCN2A*, can underlie AHC or AHC-like presentations. Functional characterization of these variants using in vitro electrophysiological assays demonstrated altered sodium channel activity: the indel variant caused loss of function, while the missense variants resulted in abnormal channel kinetics, collectively supporting their pathogenicity. Given that the *SCN2A* gene encodes the neuronal voltage-gated sodium channel Nav1.2, these findings further emphasize disrupted neuronal excitability as a key pathogenic mechanism in a subset of AHC phenotypes. Clinically, *SCN2A*-related cases often present with a combination of hemiplegic episodes, epilepsy and developmental delay, which can make differentiation from developmental and epileptic encephalopathies with paroxysmal motor features more challenging [8].

Increasing evidence suggests that the *ATP1A2* gene is a contributor to AHC in addition to its established role in familial hemiplegic migraine. The association was first reported by Bassi et al. [22] and has since been supported by further studies documenting both de novo and biallelic *ATP1A2* variants in individuals exhibiting AHC or similar clinical presentations [23,24,25,26]. Notably, the *ATP1A2* and *ATP1A3* genes encode distinct α-subunits of Na^+^/K^+^-ATPase, highlighting a potential common mechanism involving impaired ion transport and neuronal excitability in the pathogenesis of hemiplegic disorders with diverse genetic backgrounds [22,23,24,25,26].

Several additional genes have been reported more sporadically in association with AHC or AHC-like phenotypes. *CACNA1A*, another key gene in hemiplegic migraine, has been identified in a patient presenting with features overlapping hemiplegic migraine and AHC, further supporting a partial clinical and molecular overlap between these conditions [27]. The *SLC2A1* gene is particularly relevant, as GLUT1 deficiency can mimic AHC through paroxysmal motor events and hemiplegic episodes. In at least one reported case, this overlap was clinically significant enough to prompt consideration of ketogenic therapy, highlighting its diagnostic and therapeutic implications [28,29]. The *SLC1A3* gene has also been discussed in the context of AHC-like presentations, although evidence remains limited and less consistent [30].

Finally, some more recently discussed genes, such as *TBC1D24* [31] and *CLDN5* [32,33], should be interpreted with caution. Although they are relevant in the differential diagnosis of AHC or have been reported in isolated cases with AHC-like phenotypes, the current level of evidence remains limited compared to genes such as *SCN2A* or *ATP1A2*. Therefore, in a literature review, they are presented as emerging or putative contributors rather than established AHC genes. The *TBC1D24* gene encodes a protein involved in vesicular trafficking and synaptic function, and pathogenic variants in this gene are associated with a broad spectrum of epilepsy syndromes. In rare cases, patients with *TBC1D24* variants have been reported to present with paroxysmal neurological episodes and movement disorders that partially overlap with AHC, including episodic weakness or hemiplegia-like events [31]. However, these presentations are typically accompanied by prominent epilepsy and do not consistently reproduce the core clinical features of classical AHC, suggesting that *TBC1D24*-related disease represents a phenotypically overlapping but distinct entity. Similarly, *CLDN5*, which encodes a tight junction protein critical for blood–brain barrier integrity, has recently been implicated in a small number of patients with alternating hemiplegia or AHC-like presentations, often associated with additional features such as microcephaly and neurodevelopmental impairment [32,33]. The proposed pathogenic mechanism involves disruption of blood–brain barrier function, representing a pathway distinct from the ion transport and neuronal excitability mechanisms typically implicated in AHC [32]. However, given the very limited number of reported cases and the lack of replication in independent cohorts, the role of the *CLDN5* gene in AHC remains uncertain.

Taken together, these observations further support the view of AHC as part of a broader spectrum of paroxysmal neurodevelopmental disorders, characterized by shared mechanisms involving ion homeostasis, synaptic function, and cerebral energy metabolism. This is particularly relevant in the context of rare diseases with a multigenic background and an increasing number of novel gene candidates, where comprehensive genetic characterization is essential for accurate diagnosis and guiding clinical management, as demonstrated in previous studies [34,35,36].

**Table 2:** Genes associated with AHC, AHC-like phenotypes, or relevant differential diagnosis.

Gene	Functional Class	Level of Evidence in AHC Context	Typical Phenotype/Interpretation	Representative Evidence
*ATP1A3*	Na^+^/K^+^-ATPase α3	Strong	Classical AHC	[8,21]
*RHOBTB2*	Proteostasis/ubiquitination/atypical Rho GTPase	Moderate for AHC-like/AHC-spectrum phenotype	DEE with paroxysmal movement disorder and hemiplegia-like episodes	[8,9,14]
*SCN2A*	Voltage-gated sodium channel (Nav1.2)	Strong in ATP1A3-negative AHC/AHC-like cases	AHC or AHC-like phenotype with epilepsy/DEE	[8]
*ATP1A2*	Na^+^/K^+^-ATPase α2	Moderate	AHC–hemiplegic migraine overlap	[22,23,24,25]
*CACNA1A*	Voltage-gated calcium channel	Limited	Atypical AHC or hemiplegic migraine overlap	[27]
*SLC2A1*	Glucose transporter (GLUT1)	Limited	AHC mimic/overlap phenotype	[28,29]
*SLC1A3*	Glutamate transporter (EAAT1)	Limited	Overlap phenotype with episodic ataxia/hemiplegic features	[30]
*TBC1D24*	Vesicular trafficking/epilepsy-related protein	Emerging	AHC-like phenotype	[31]
*CLDN5*	Blood–brain barrier tight junction protein	Emerging	Alternating hemiplegia/AHC-like presentation (often with microcephaly)	[32,33]

### 5.2. RHOBTB2-Phenotypes—Complex Neurodevelopmental Disorders Fulfilling the Criteria for AHC

Pathogenic variants in the *RHOBTB2* gene have just recently been established as causes of a rare type of neurodevelopmental disorder with a diverse phenotype. To date, fewer than 100 cases of *RHOBTB2*-related disorder have been documented worldwide [18]. The first reports on *RHOBTB2* pathogenic variants included patients with de novo missense variants grouped in the BTB domain region, who exhibit a relatively homogeneous phenotype including severe DEE, early-onset epilepsy, intellectual disability and movement disorders [12,17]. These variants were shown to have a gain-of-function effect, and several were recurrent, with the variant detected in our patient being among them [12,37,38]. Nevertheless, studies also reported patients with missense variants clustered in the GTPase domain who showed somewhat milder and more diverse phenotypes, with one patient presenting paroxysmal movement disorders alone at early adolescence [39,40]. Moreover, a recent study described patients with variable neurodevelopmental phenotypes (including intellectual disability and seizures) who were found to have biallelic loss-of-function variants in *RHOBTB2* [18]. The latest study further demarcated these different types of variants in a human induced pluripotent stem cell setting, revealing markedly altered neuronal activity and excitability due to BTB-domain variants but not GTPase-domain variants or total loss of *RHOBTB2* [41]. Up until then, *RHOBTB2* was only linked to autosomal dominant inheritance, so this discovery increases the knowledge of this gene in cases of autosomal recessive inheritance.

The study by Zagaglia et al. [9] was the first to reveal the link between the *RHOBTB2* gene and AHC, thus significantly widening the previously described phenotypic spectrum. They pinpointed a complex polymorphic movement disorder with paroxysmal elements resembling AHC. All (11) patients harbored heterozygous missense variants involving exon 9 of the *RHOBTB2* gene, which were confirmed to be de novo in nine. While all reported patients presented with a complex movement disorder phenotype, many of them fulfilled the criteria for AHC, where ten patients presented with paroxysmal elements, and eight had hemiplegic episodes. All patients had intellectual disability, while seven of them had epilepsy [9].

Ever since, it has been recognized that AHC phenotypes caused by *RHOBTB2* pathogenic variants differ significantly, thus posing an intriguing question as to whether they could be acknowledged as typical AHC or AHC-like. In the past several years, a number of studies and case reports have described patients with various forms of paroxysmal attacks, which included hemiplegia, as well as other clinical manifestations. A comprehensive study by De Pedro Baena et al., 2023 [14], included seven patients with *RHOBTB2*-related disorders, of which one could be interpreted as an AHC-like phenotype, presenting with seizures, paroxysmal ataxia and diplegia, as well as stereotypies. In a search for novel causative genes for AHC apart from *ATP1A3*, Panagiotakaki described a patient with quadriplegia and paroxysmal attacks who met the criteria for AHC, thus referred to as typical AHC [8]. Two recent case reports of AHC-like phenotypes further expand the *RHOBTB2* spectrum, where Innocenti et al. [42] presented a patient with hemiplegic migraine in addition to paroxysmal attacks, while Hoang et al. [43] documented an interesting case of abrupt and severe onset of dystonia, chorea and hemiplegic episodes in a pediatric patient.

On the other hand, as pointed out by Mikati et al. [5], not every early-onset alternating hemiplegia is alternating hemiplegia of childhood. Our patient complied with the criteria for the diagnosis of AHC according to the Revised Aicardi Criteria Proposal-2021. Both essential criteria were met: recurrent paroxysmal episodes of hemiplegia alternating between sides and/or quadriplegia, as well as evidence of underlying abnormal neurological development. Also, four of the five major criteria were fulfilled: age of onset prior to 18 months, episodes of dystonia, occurrence of multiple episode types either independently or concurrently within a single episode with evolution from one symptom to another, and paroxysmal ocular manifestations including upward gaze and nystagmus. Notably, the major criterion of a confirmed pathogenic variant in the *ATP1A3* gene was not fulfilled, along with no consistent improvement in hemiplegic episodes with sleep. Furthermore, four minor criteria were met: epileptic seizures occurring in isolation or in combination with other paroxysmal events; episodes of altered consciousness of non-epileptic origin, either isolated or concurrent with other spells; abnormal motor function characterized by severe tonic episodes with dystonic posturing, including bulbar dystonia; and episodes of autonomic dysfunction, manifested by febrile episodes without identifiable infectious etiology. According to revised diagnostic criteria for AHC, confirmation of diagnosis requires fulfillment of two essential criteria, three major criteria or two major criteria and three minor criteria. Our patient met both essential criteria, four major criteria and four minor criteria, thereby confirming the diagnosis of AHC. A summary of AHC phenotypes (with further clinical manifestations) and identified genetic variants in the *RHOBTB2* gene for previously reported patients, along with a patient from this study, is presented in Table 3.

Pathogenic variants affecting the BTB domain of RHOBTB2, and particularly those involving codon 511, have been predominantly associated with a DEE phenotype, in which early-onset seizures and severe neurodevelopmental impairment represent the hallmark features. Although paroxysmal events, including hemiplegia, are frequently observed in this patient group [13,37], the presence of AHC-like features does not necessarily imply fulfillment of the formal diagnostic criteria for AHC. Indeed, variant c.1532G>A (p.Arg511Gln) was described as disease-causing in the very first report in which the *RHOBTB2* gene was linked to the development of DEE [12]. This variant affects the arginine residue located at the dimer interface of the second BTB domain. This residue is recurrently affected, and apart from a change to glutamine, changes to tryptophan and glycine were also observed [9,37]. Given that pathogenic missense variants often cluster in hotspots and essential protein domains, Arg511 was recurrently affected along with the asparagine at position 510 [12,18]. Our patient presented with a rather severe phenotype in line with previously described cases with pathogenic de novo missense variants in the BTB domain. Four other patients with pathogenic variants affecting the Arg511 residue who presented with AHC were reported to have seizures, ataxia, dyskinesia, dystonia, head and eye deviation, as well as possible encephalopathic episodes [9]. Our patient exhibited developmental delay with hypotonia during infancy, while her first episode requiring hospitalization occurred at the age of 17 months. In addition to alternating hemiplegia and other paroxysmal episodes, seizures comprised part of her clinical presentation, including generalized tonic clonic seizures, as well as a severe status epilepticus that lasted for 2 h. The variety of seizures is common for *RHOBTB2* patients, and the majority of the patients exhibit a diverse range of seizures, which include generalized tonic clonic seizures, febrile seizures, focal seizures, tonic seizures, and myoclonic seizures, and also in many of them, status epilepticus. Status epilepticus, designated as an acute, prolonged epileptic crisis, was a prevalent experience for *RHOBTB2* patients since it was reported in more than half of them [40]. In some patients, the seizures were refractory, but for the majority, a good response to treatment was achieved after multiple antiepileptic drugs. Seizures of certain patients were controlled by carbamazepine, while others responded better to levetiracetam, but studies report the use of a variety of drugs, such as phenobarbital, lamotrigine, topiramate, clobazam, divalproex sodium, and zonisamide, and even the ketogenic diet, whereas corticosteroids (methylprednisolone) were effective in treating acute encephalopathy episodes [12,14].

Nevertheless, it is worth emphasizing that differentiating between seizures and paroxysmal events within AHC presents a considerable challenge, especially given that epileptic seizures may occur at the same episode as hemiplegia. In our patient, clinical diagnosis was established following the second episode of paroxysmal hemiplegia involving the contralateral side of the body, along with impairment of consciousness. In a number of her hemiplegic episodes, seizures did constitute part of the clinical presentation, as documented by ictal EEG recordings. The most recent tonic episode was particularly severe, characterized by generalized rigidity, dystonic upward gaze deviation and a life-threatening airway spasm requiring intubation and mechanical ventilation for a period of three days. Even though such tonic episodes might mimic epileptic events, ictal EEG recordings demonstrated an absence of epileptic discharges. Continuous midazolam infusion may represent an effective therapeutic option in the management of severe tonic and dystonic episodes in patients with AHC.

Following fulfillment of AHC criteria, the confirmation of the diagnosis of AHC for our patient prompted the inclusion of flunarizine in combination with antiseizure medication. The introduced therapeutic regimen combining levetiracetam and carbamazepine demonstrated efficacy in achieving seizure control without observed side effects. Over the subsequent three-year follow-up period, the patient maintained remission with good seizure control and, probably due to treatment with flunarizine, without episodes of hemiplegia. While complete recovery has been achieved in the gross motor domain, persistent aphasia and intellectual disability remain the neurological hallmarks of our patient.

Given that the published literature on AHC associated with pathogenic variants in the *RHOBTB2* gene is rather scarce, it is difficult to ascertain whether this type of the disease carries a more severe clinical presentation compared to AHC associated with *ATP1A3* variants. The present case report aims to contribute to the existing knowledge by suggesting that AHC caused by pathogenic variants in the *RHOBTH2* gene may have a particularly severe clinical presentation. Further studies encompassing larger cohorts of *RHOBTB2*-related AHC patients are needed to elucidate whether this hypothesis can be corroborated.

### 5.3. RHOBTB2-Related Disorders: Mechanism Behind the Complex Phenotype

The first studies on the RHOBTB2 protein were conducted more than two decades ago, when it was known as a tumor suppressor by the name of DBC2 [11]. This protein was shown to belong to the RhoBTB subfamily of small atypical Rho GTPases, a group of proteins characterized by a multidomain architecture that combines a Rho-like GTPase domain with two BTB domains and a conserved C-terminal region. The BTB domains were proven to play a central role in the function and regulation of RHOBTB2 by mediating protein–protein interactions and thus allowing the formation of both homodimers and heterodimers with other members of the RhoBTB protein family [44]. In addition to mediating intermolecular interactions, the BTB domains can interact intramolecularly with the GTPase domain of RHOBTB2, contributing to the maintenance of its auto-inhibited conformation. This domain organization allows RHOBTB2 to function both as a signaling molecule and as an adaptor protein within ubiquitin-mediated protein degradation pathways, as the BTB domains also enable RHOBTB2 to interact with the ubiquitin ligase scaffold protein Cullin-3 (CUL3), linking RHOBTB2 to the CUL3-dependent ubiquitin ligase complex [44]. Through this interaction, RHOBTB2 can function as a substrate adaptor, recruiting proteins to the ubiquitination machinery. Remarkably, RHOBTB2 itself can undergo ubiquitination within this complex, leading to its degradation by the proteasome. This auto-ubiquitination mechanism creates a self-limiting regulatory loop that controls intracellular RHOBTB2 levels, as it was found that mutant RHOBTB2 proteins exhibit impaired interaction with CUL3, resulting in reduced ubiquitination and proteasomal degradation of RHOBTB2 itself [44,45,46]. Such impairment could lead to increased intracellular levels of RHOBTB2 and altered regulation of downstream cellular pathways.

Upon the first association of RHOBTB2 and neurodevelopmental disorders, Straub et al. [12] further elucidated the role of RHOBTB2 in neurological function in a Drosophila model, where they indeed found that elevated amounts of the Drosophila ortholog RhoBTB led to seizures and locomotor defects. Nevertheless, they found that the pathophysiological mechanism may be more complex than the simple disruption of CUL3 ubiquitin ligase binding, as they did not discover a significant reduction in binding between mutant RHOBTB2 proteins and CUL3. Several disease-associated RHOBTB2 missense variants (including recurrently affected arginine at position 511) residing in the BTB domain were investigated to determine whether they impair interaction with CUL3. Even though some of the variants lie outside the predicted binding interface and therefore would not be expected to directly disrupt the interaction, it was demonstrated that these variants may still interfere with normal ubiquitination and degradation of RHOBTB2. These variants may compromise proteasomal degradation of RHOBTB2 despite a preserved interaction with CUL3, possibly through altered stability of the BTB domain or impaired dimer formation [12]. As a consequence, mutant RHOBTB2 proteins may accumulate in the cell. Increased levels of RHOBTB2 and potentially other RHOBTB2-dependent substrates could therefore contribute to disease pathogenesis. Although elevated RHOBTB2 levels could not be directly confirmed in patient-derived samples, experimental data from cellular models support this possibility, and dominant-negative or gain-of-function effects of the variants cannot be excluded [12].

**Figure 4:**
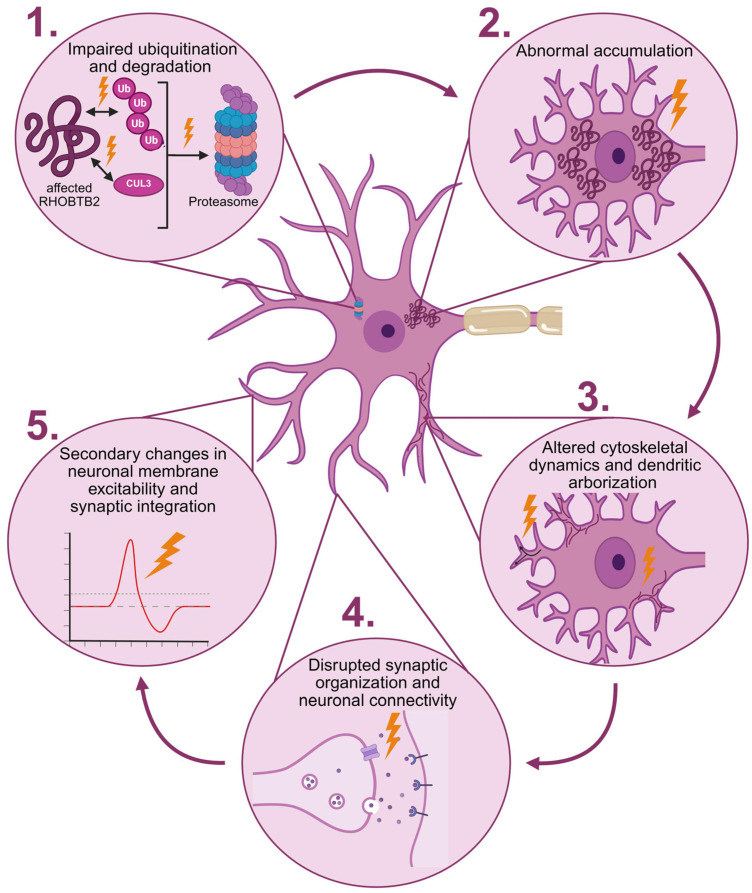
Proposed downstream cascade linking an altered RHOBTB2 protein and impaired neuronal function in *RHOBTB2*-related AHC. Impaired ubiquitination and degradation of an altered RHOBTB2 protein (1) can lead to its abnormal accumulation in the cytoplasm (2). This can, in turn, result in altered cytoskeletal dynamics and dendritic arborization during neuronal development (3), leading to disrupted synaptic organization and neuronal connectivity (4). As a consequence, secondary changes in neuronal membrane excitability and synaptic integration may arise (5), outlining *RHOBTB2*-associated AHC clinical presentation. Created in BioRender. Stankovic, S. (2026) https://BioRender.com/2uq2f3g (accessed on 27 April 2026).

Within this framework, pathogenic variants affecting the BTB domain of RHOBTB2 may disrupt the self-limiting regulatory loop that normally controls RHOBTB2 stability. Impaired ubiquitination and degradation may result in abnormal accumulation of the RHOBTB2 protein. Elevated RHOBTB2 levels could, in turn, alter cytoskeletal dynamics and dendritic arborization during neuronal development, processes in which RHOBTB2 has been implicated [9]. Alterations in dendritic architecture may disrupt synaptic organization and neuronal connectivity, leading to secondary changes in neuronal membrane excitability and synaptic integration (Figure 4) [47]. Consistent with this hypothesis, electrophysiological studies in human neuronal models provide evidence of altered neuronal activity associated with RHOBTB2 variants. In experiments using a standard hiPSC (human induced Pluripotent Stem Cell) line differentiated into neuronal progenitor cells and neurons, introduction of BTB-domain missense variants using CRISPR/Cas9 resulted in increased firing frequency, prolonged action potential half-width, and reduced depolarization speed, indicating abnormal neuronal excitability [41]. While these findings were derived from the existing literature, we linked the individually reported effects of pathogenic RHOBTB2 variants into a unified pathogenic framework: impaired ubiquitin-mediated degradation leads to RHOBTB2 accumulation, which disrupts cytoskeletal and dendritic organization during development, ultimately giving rise to secondary changes in neuronal excitability. This hypothesis remains to be fully elucidated in future studies.

Building on this proposed model, we further hypothesize that this mechanism may distinguish *RHOBTB2*-associated disorders from other forms of AHC, which result from pathogenic variants in genes encoding ion channels, proteins regulating channel function, or transporters. Indeed, SCN2A and CACNA1A are voltage-gated ion channels, while ATP1A2 and ATP1A3 are subunits of the Na^+^/K^+^-ATPase pump, so they directly affect neuronal membrane excitability and action potential generation. In contrast, *RHOBTB2*-related diseases may arise primarily from disturbances in neuronal structural organization, cytoskeletal dynamics, and synaptic architecture. In this model, neuronal network instability and altered excitability may occur as secondary consequences of disrupted neuronal development and connectivity rather than from primary defects in ion channel/transporter structure. In this setting, *RHOBTB2*-associated disease may represent a disorder of neuronal structural and synaptic regulation in which hyperexcitability arises as a downstream consequence of impaired ubiquitin-mediated protein turnover during brain development.

Nevertheless, the complex interplay between AHC and DEE phenotypes, often in patients with the same genetic variants, remains an open question. The molecular mechanism underlying a particular AHC phenotype in patients with pathogenic *RHOBTB2* variants is still incompletely understood. The variants identified in AHC cases span multiple functional domains of the protein, with the majority clustering within the BTB domains, critical for CUL3-mediated ubiquitination and protein–protein interactions [9,43]. The distinction between *RHOBTB2*-associated AHC and the more frequently reported developmental and epileptic encephalopathy phenotype highlights a significant gap in our understanding of how *RHOBTB2* dysfunction leads to hemiplegic episodes. Transcriptomic profiling in human-based neuronal models, such as neuronal cell lines, patient-derived iPSC neurons or organoids, has made a great impact by illuminating different neurodevelopmental diseases [48,49]. This approach could therefore offer valuable insight into the downstream consequences of *RHOBTB2* dysfunction in the context of AHC. Single-cell RNA sequencing could help characterize cell-type- and region-specific expression of *RHOBTB2* across different stages of neuronal development, potentially identifying neuronal populations with higher vulnerability, while differential gene expression analysis in cells carrying pathogenic variants could further elucidate downstream transcriptional consequences [41]. Furthermore, comparison of transcriptomic signatures between *RHOBTB2*-related AHC and *ATP1A3*-related AHC could reveal shared molecular substrates that may explain similar clinical presentations. Given that *RHOBTB2*-associated AHC is extremely rare, hindering large-scale patient cohort studies, functional genomic approaches like this may represent one of the most feasible ways to advance in understanding this condition.

Rare monogenic disorders with complex phenotypes have long posed a scientific puzzle, with a tendency to be compared to complex diseases due to their presentation as a spectrum of disease phenotypes. Even though the disease-causing variants stand as the main determinant of a corresponding phenotype, inconsistencies in genotype–phenotype correlation have been noted for many rare monogenic disorders [50,51]. The reports on patients (even siblings) bearing the same variants but presenting with different clinical manifestations, along with healthy individuals proven to harbor pathogenic variants in genes for well-known Mendelian disorders, have shifted our understanding of simple monogenic disorders to a rather complex disease phenotype [52,53,54]. The search for additional genetic factors that contribute to the final *RHOBTB2* phenotype could not only shed more light on the molecular mechanisms underlying this complex neuropathology but also pave the way for research on novel therapeutics for individualized treatment of patients presenting with *RHOBTB2*-related AHC.

## 6. Conclusions

According to the clinical course and the results of our study, we conclude that the described patient has a severe phenotype of alternating childhood hemiplegia associated with the *RHOBTB2* gene variant, associated with developmental delay, aphasia and epilepsy. This is the first case of a patient with AHC caused by a pathogenic variant in the *RHOBTB2* gene in Serbia. Variants in the *RHOBTB2* gene should be considered as disease-causing in patients with early-onset seizures, delayed psychomotor development and alternating hemiplegia of childhood. Based on the currently available cases, including those reported in the literature and the herein presented case, our findings suggest that *RHOBTB2*-related AHC may be associated with a more severe clinical presentation than ATP1A3-related AHC. The growing number of cases with *RHOBTB2*-related disorders continues to expand the spectrum of associated phenotypes and contribute to the much-needed knowledge on this ultra-rare disease.

## Figures and Tables

**Table 1:** Number of articles retrieved per search term in each database.

Search Term	PubMed	Google Scholar	Europe PMC
*RHOBTB2* neurodevelopmental	11	268	81
*RHOBTB2* paroxysmal	7	104	46
*RHOBTB2* dyskinesia	5	95	35
*RHOBTB2* hemiplegia	4	71	33
*RHOBTB2* hemiplegic episode	3	48	12
*RHOBTB2* DEE	4	79	29
*RHOBTB2* alternating hemiplegia of childhood	2	61	26
*RHOBTB2* AHC	2	30	18
*RHOBTB2* AHC-like	2	6	17

**Table 3:** Summary of genotype, phenotype and additional clinical characteristics of previously reported patients with *RHOBTB2*-related AHC.

Variant in RHOBTB2 cDNA (NM_001160036.1)	Variant in RHOBTB2 Protein	Number of Affected Patients	Inheritance	ACMG Classification	AHC Clinical Phenotype	Other Clinical Characteristics	Resource
c.103G>A	p.Glu35Lys	1	de novo	Likely Pathogenic	typical AHC	Quadriplegia, paroxysmal attacks	[8]
c.342C>G	p.Asp114Glu	1	de novo	Likely Pathogenic	AHC-like	Dyskinesia (paroxysmal), dystonia (paroxysmal), tongue protrusion, cerebellar dysgenesis	[43]
c.359G>A	p. Gly120Glu	1	de novo	Likely Pathogenic	AHC-like	Seizures, ataxia (paroxysmal), diplegia (paroxysmal), stereotypies	[14]
c.717G>C	p.Trp239Cys	1	de novo	Likely Pathogenic	AHC-like	Encephalopathic episode, status epilepticus, dyskinesia (baseline and paroxysmal), dystonia (baseline and paroxysmal), nystagmus	[9]
c.722C>A	p.Ser241Tyr	1	de novo	Likely Pathogenic	AHC-like	Encephalopathic episode, dyskinesia (baseline), dystonia (paroxysmal), myoclonus, stereotypies	[9]
c.722C>T	p.Ser241Phe	1	de novo	Likely Pathogenic	typical AHC	Quadriplegia, paroxysmal attacks	[8]
c.1448G>A	p.Arg483His	1	de novo	Pathogenic	AHC-like	Dystonia (paroxysmal), head and eye deviation	[9]
c.1519C>T	p.Arg507Cys	1	unconfirmed	Pathogenic	AHC-like	Seizures, possible dystonia	[9]
c.1520G>T	p.Arg507Leu	1	de novo	Likely Pathogenic	AHC-like	Encephalopathic episode, right hemisphere cortical atrophy, right hemispheric hypoperfusion, paroxysmal attacks, hemiplegic migraine	[42]
c.1531C>G	p.Arg511Gly	1	de novo	Likely Pathogenic	AHC-like	Electrographic seizures, mild static cerebellar atrophy, possible encephalopathic episode, ataxia (baseline and paroxysmal), stereotypies	[9]
c.1531C>T	p.Arg511Trp	1	de novo	Pathogenic	AHC-like	Seizures, encephalopathic episode, dystonia (baseline and paroxysmal), ataxia (paroxysmal), tremor myoclonus	[9]
c.1532G>A	p.Arg511Gln	3	de novo (all)	Pathogenic	typical AHC; AHC-like	Multifocal discharges, thin corpus callosum, possible encephalopathic episode, dyskinesia (basal and paroxysmal), dystonia (paroxysmal), head and eye deviation, tongue protrusion	[9]
						Seizures, status epilepticus, possible encephalopathic episode, dyskinesia (basal and paroxysmal), dystonia (paroxysmal), head and eye deviation	
						Seizures, status epilepticus, nystagmus, eye deviation, reduction in supratentorial white matter volume, zones of dysmielinization, hypotonia, quadriplegia	This study

## Data Availability

The original contributions presented in this study are included in this article. Further inquiries can be directed to the corresponding author.

## Supplementary Materials

The following supporting information can be downloaded at https://www.mdpi.com/article/10.3390/diseases14050166/s1. Figure S1: Electropherograms showing the *RHOBTB2* variant c.1532G>A (p.Arg511Gln); Figure S2: Stacked bar plots showing the number of publications across 5 categories; Figure S3: Flow diagram representing the literature search process.

## Abbreviations

The following abbreviations are used in this manuscript:

AHC	Alternating Hemiplegia of Childhood
RhoBTB2	Rho-Related BTB Domain-Containing Protein 2
DEE	Developmental and Epileptic Encephalopathy
DBC2	Deleted in Breast Cancer 2
CT	Computerized Tomography
MRI	Magnetic Resonance Imaging
EEG	Electroencephalography
WES	Whole Exome Sequencing
ACMG	American College of Medical Genetics and Genomics
hiPSC	Human Induced Pluripotent Stem Cell
