# Supplementary material for: RHOBTB2-Associated Neurological Phenotypes and Underlying Mechanisms: Alternating Hemiplegia of Childhood Beyond ATP1A3
Source: Diseases. 2026 May 9;14(5):166. doi: 10.3390/diseases14050166 (PMC13205392; doi:10.3390/diseases14050166)
Supplement: Supplementary file 1 [file diseases-14-00166-s001.zip › diseases-4314785-supplementary.pdf]

SUPPLEMENTARY MATERIALS

RHOBTB2-Associated Neurological Phenotypes and Underlying Mechanisms: Alternating Hemiplegia of Childhood Beyond *ATP1A3*

Ruzica Kravljanac[1,2,†], **Kristel Klaassen**[3,*,†], **Vladimir Oparnica**[1], **Biljana Vucetic Tadic**[1,2], **Marina Andjelkovic**[3], **Anita Skakic**[3], **Sara Stankovic**[3], **Maja Stojiljkovic**[3]

[1] Institute for Mother and Child Healthcare of Serbia "Dr Vukan Cupic", 11070 Belgrade, Serbia.

[2] Faculty of Medicine, University of Belgrade, 11000 Belgrade, Serbia.

[3] Institute of Molecular Genetics and Genetic Engineering, University of Belgrade, 11042 Belgrade, Serbia

* Corresponding author: Kristel Klaassen, PhD

Institute of Molecular Genetics and Genetic Engineering, University of Belgrade

Vojvode Stepe 444a, 11042 Belgrade, SERBIA

Email: kristel.klaassen@imgge.bg.ac.rs

[†] These authors contributed equally to this work

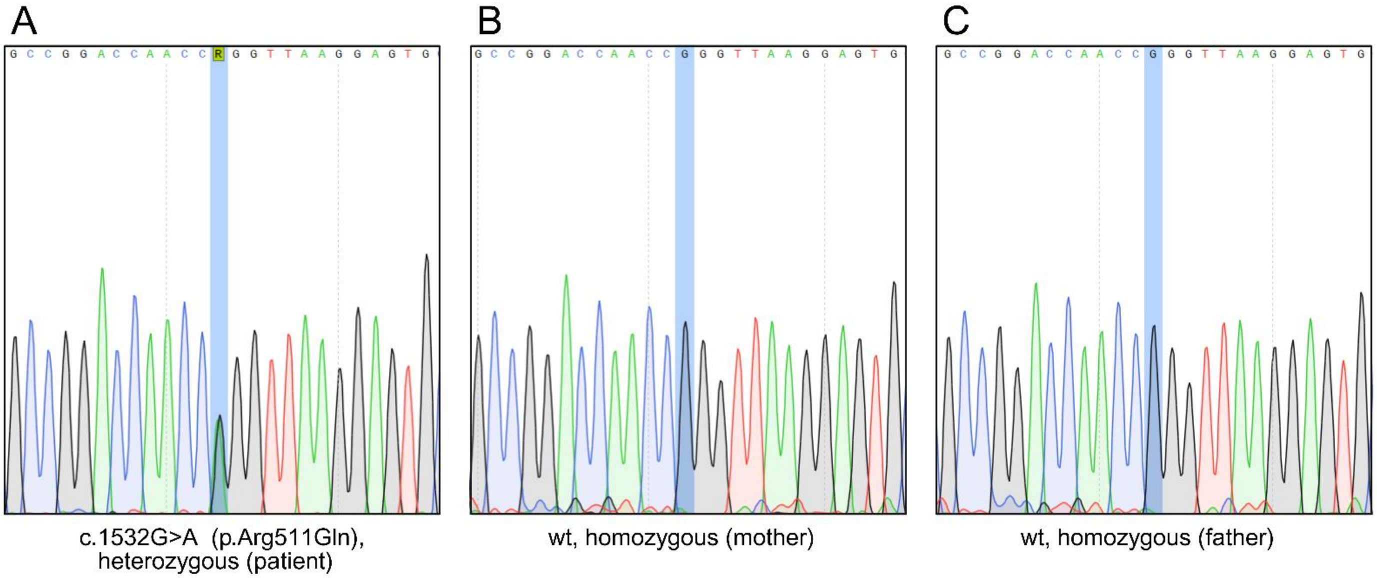

Figure S1. Electropherograms showing the *RHOBTB2* variant c.1532G>A (p.Arg511Gln). Sanger sequencing confirmed the presence of the variant c.1532G>A (p.Arg511Gln) in heterozygous state in the patient (A), while both parents demonstrated a *wild-type* genotype (B, C).

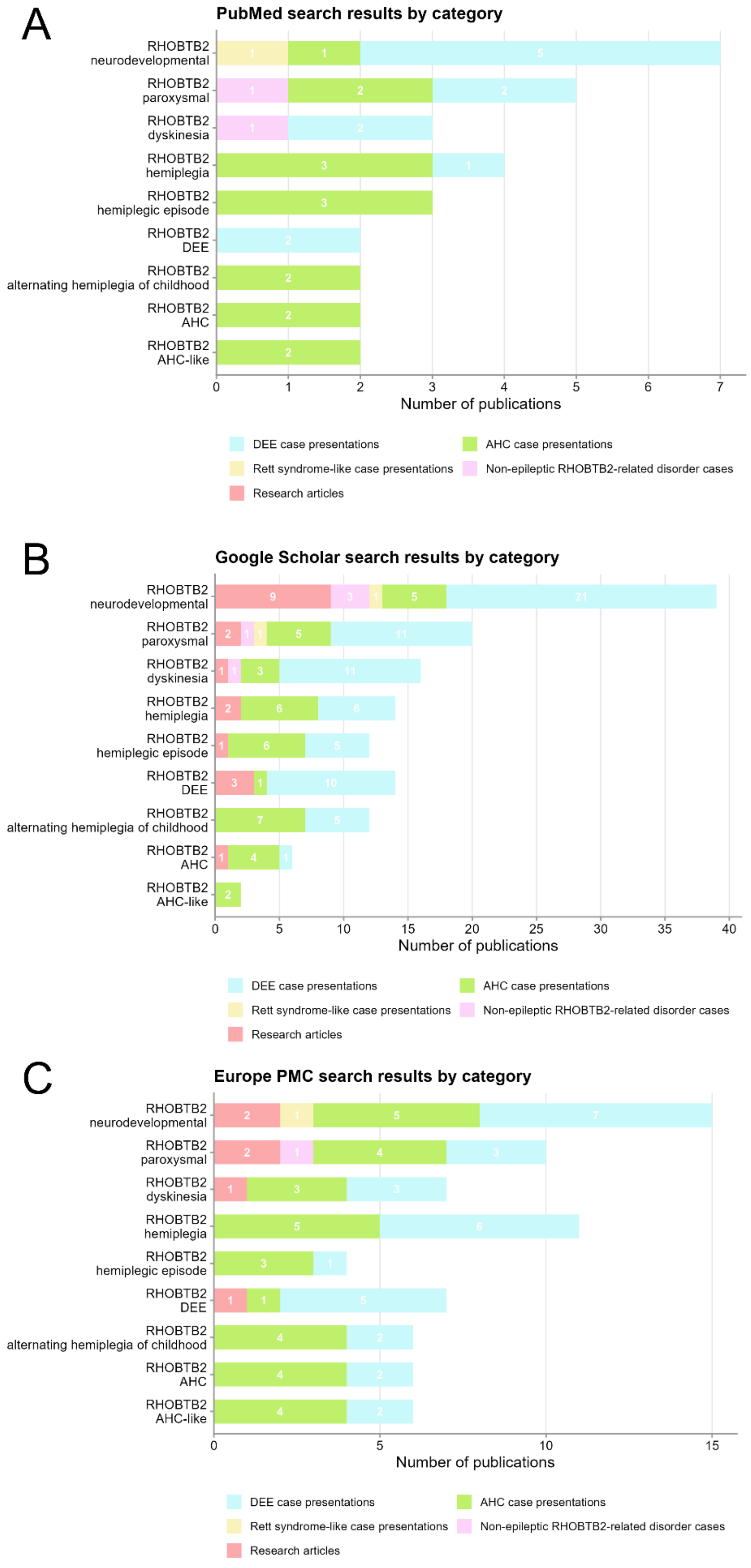

Figure S2. Stacked bar plots showing the number of publications across 5 categories. The number of publications per search term, belonging to each category is indicated by different colors. Results from 3 databases are shown: PubMed (A), Google Scholar (B), and Europe PMC (C).

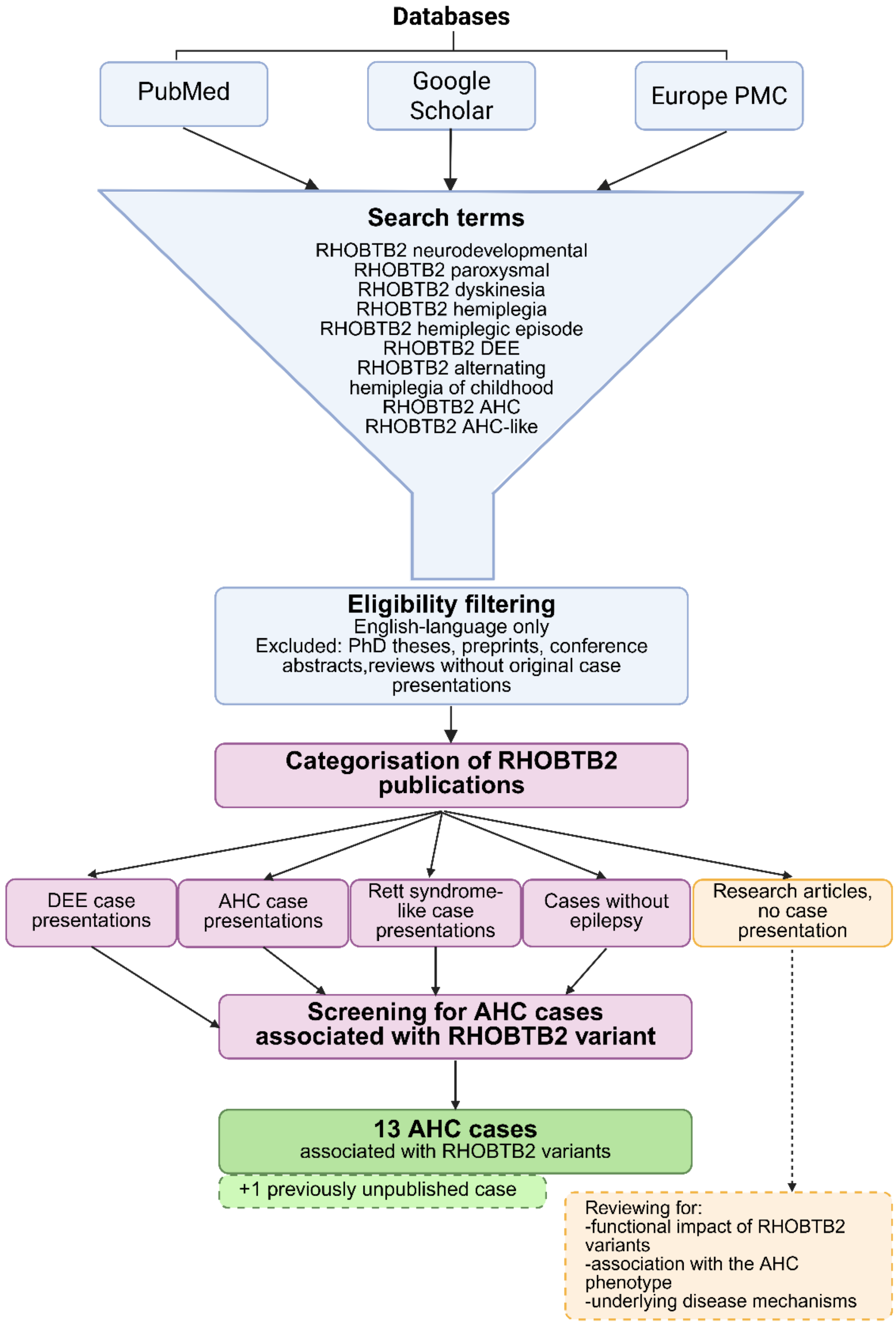

Figure S3. Flow diagram representing the literature search process. Same search terms were applied across 3 databases. Resulting publications were filtered and categorized into 5 groups. Articles containing case presentations were screened for *RHOBTB2*-associated AHC, yielding 13 published cases included in this review, along with 1 novel, previously unpublished case.
